# Low and High Birth Weight in a Hospital Population in Qassim, Saudi Arabia: An Analysis of Clinical Cutoff Values

**DOI:** 10.3390/children11121483

**Published:** 2024-12-04

**Authors:** Ashwaq Al Eed, Abdullrahman Alsalamah, Abdullah Al-Nafeesah, Osama Al-Wutayd, Rola Yousef Alzain, Ishag Adam

**Affiliations:** 1 Department of Pediatrics, College of Medicine, Qassim University, Buraydah 52571, Saudi Arabia; a.alnafeesah@qu.edu.sa; 2 Department of Pediatrics, King Saud Hospital, Unaizah 56437, Saudi Arabia; abdullrahmanha@moh.sa.gov; 3 Department of Family and Community Medicine, College of Medicine, Qassim University, Buraydah 52571, Saudi Arabia; o.alwutayd@qu.edu.sa; 4 Clinical Pharmacist, Department of Quality Assurance, Saudi Pharmaceutical Industries & Medical Appliances Corporation, Burydah 20001, Saudi Arabia; 5 Department of Obstetrics and Gynecology, College of Medicine, Qassim University, Buraydah 52571, Saudi Arabia; ia.ahmed@qu.edu.sa

**Keywords:** low birth weight, high birth weight, male, female, newborn

## Abstract

Background: Establishing cutoff values for low birth weight (LBW) and high birth weight (HBW) is crucial for clinical practice. This study aimed to determine these values in Saudi Arabia. Method: A retrospective study in 2022 analyzed the birth weights of 1605 neonates. The 10th and 90th percentiles were calculated to define LBW and HBW. Results: LBW was defined as <2450 g (10th percentile), with an incidence of 10.5%. HBW was >3650 g (90th percentile), with a prevalence of 10.3%. Comparing the WHO’s LBW definition (<2500 g) revealed significant differences but high agreement (kappa = 0.962). HBW had a prevalence of 2.4% using a 4000 g cutoff, with low agreement (Kappa = 0.357). Conclusions: This study suggests an LBW cutoff at 2450 g and an HBW cutoff at 3650 g in Saudi Arabia. A birth weight range of 2450–3650 g is considered normal, reducing unnecessary healthcare interventions.

## 1. Introduction

The birth weight of the newborn is the first weight, regardless of gestational age. It has been agreed that normal birth weight is between the 10th and 90th percentiles, serving as the lower and upper limits/cutoff, respectively; these are considered normal. On the other hand, low birth weight (LBW) is defined by the World Health Organization (WHO) as a weight of less than 2500 g [1]. A newborn is considered to have a high birth weight (HBW) if their birth weight is more than 4000 g. However, a birth weight greater than 4500 g is sometimes considered an HBW and is assumed to correspond to the 90th percentile cutoff [2,3].

Birth weight data are accessible and recorded and may be a suitable indicator of other factors associated with mortality and adverse outcomes related to the global impact of short- and long-term consequences. Both LBW and HBW are associated with several adverse maternal, perinatal, and neonatal health effects [4,5,6]. LBW is a global health issue, with 20.5 million LBW newborns/babies in the world [7]. This represents considerable variation in the prevalence of LBW among the many affected countries [8]. A recent (2024) study has shown that 153 (8.2%) of 1855 newborns in Riyadh, Saudi Arabia were LBW deliveries [9]. LBW is a known predictor of neonatal mortality and morbidity; the late development of non-communicable diseases, such as diabetes and cardiovascular diseases, has been reported [8,10].

HBW is a global health problem whose prevalence varies in different countries. For example, 7.6% was reported in China and 9% in the USA. In contrast, in sub-Saharan countries, 9–14% of neonates are HBW [11,12,13,14]. It has recently been (2024) shown that 37 (2.0%) of 1855 newborns in the capital Riyadh, Saudi Arabia were macrosomic [9]. Several adverse perinatal effects, such as perinatal death, birth trauma, shoulder dystocia, clavicular fracture, and brachial plexus injuries, have been attributed to HBW [2].

Traditionally, health professionals consider birth weights between 2500 and 4000/4500 g normal. However, these figures were assumed to depend on data derived primarily from developed and affluent countries [15,16]. Therefore, applying these values and their convenience for worldwide clinical use is questionable. Thus, the WHO urges communities to develop their own reference values and cutoffs for birth weight that could be used in clinical practice. Previously, the traditional use of the WHO cutoff (2500 g) for LBW as an epidemiological tool assumed that newborns weighing less than 2500 g were 20 times at higher risk of dying than newborns who weighed more than 2500 g [1]. Moreover, a 30.0% reduction in LBW (using the traditional cutoff or 10th percentile) is one part of the plan for maternal, infant, and young child nutrition in the global nutrition targets for 2025 [8].

Having a specific reference value for LBW and HBW will precisely detect the infants needing health services and can help avoid unnecessary hospital care and admission. Hence, it could reduce the cost of health services. Few previous studies have been conducted in developing countries to access LBW and HBW cutoff values [17,18,19]. Several factors, such as gestational hypertension and lower gestational age, were associated with LBW in Saudi Arabia [9,20]. However, to our knowledge, there is no published data on the clinical cutoff for both LBW and HBW in Saudi Arabia. This study was, therefore, conducted to determine the cutoff values for both LBW and HBW in King Saud Hospital-Unaizah, Qassim, Saudi Arabia.

## 2. Materials and Methods

### 2.1. Study Setting, Duration, Population, and Sampling

A retrospective study was conducted at the maternity and pediatric Department of King Saud Hospital-Unaizah, Qassim, Saudi Arabia, from January to December 2022. Al-Qassim is in the heart of the country (Saudi Arabia), near the geographic center of the Arabian Peninsula. Al-Qassim has a population of 1,336,179 and an area of 58,046 km^2^. The hospital is located in Unaizah City, the second largest city in the Al-Qassim region, with a population of 184,600. In this study, strengthened reporting of observational studies in epidemiology (STROBE) guidelines was strictly followed [21]. King Saud Hospital-Unaizah is the second largest hospital (Buraidah Maternity and Children’s Hospital is the largest one) in AL-Qassim, Saudia Arabia. The maternity unit is in a secondary hospital and conducts standard and cesarean deliveries for women. The hospital services include an emergency department, an outpatient department, pediatrics, and neonatal units. Teams of obstetricians and pediatricians care for patients in maternity and pediatric units, respectively, including a neonatal unit. All deliveries in Saudi should be in the hospitals free of charge. Home deliveries are not allowed in Saudi Arabia. Two female doctors reviewed the medical records of the women in the obstetrics department. The collected information included the age, parity, birth weight, and sex of the newborn.

### 2.2. Inclusion and Exclusion Criteria

Live, singleton hospital deliveries whose birth weight ≥1000 g and gestational age ≥28 weeks were eligible for inclusion in this study. Data on deliveries before 28 weeks of gestation, dead neonates, multiple deliveries, birth weight less than 1000 g, and neonates with congenital malformations were excluded.

### 2.3. Data Collection and Variables

In this hospital, the baby’s weight was measured to the nearest gram using the standard method (SECA 374 Baby scale, electronic, 20 kg, Hamburg, Germany) and calibrated daily.

### 2.4. Sample Size

A sample size of 1605 neonates was calculated depending on the reports on the previous prevalence (14.0–15.0%) of LBW in different regions of Saudi Arabia [22]. We assumed that 19% vs. 13.0% was the expected percentage of women with age ≥35 years who delivered LBW babies and women who delivered normal-weight babies. As mentioned above, this assumption was based on previous work in other parts of Saudi Arabia [22]. This sample size has a power of 80% and Type I error (i.e., *p*-value < 0.05).

### 2.5. Statistical Analysis

The data were double-checked, entered, and then analyzed using the Statistical Program for the Social Sciences (SPSS) software, version 22 (SPSS Inc., Chicago, IL, USA). Categorical variables were expressed as proportions, frequencies, and percentages. Continuous variables (maternal age, parity, and birth weight) were checked for normality using the Shapiro–Wilk test and were not normally distributed. Thus, the median interquartile range (IQR) was used to express the data. The 10th and 90th percentiles of birth weight were calculated, and their values were the cutoff for LBW and HBW, respectively. After that, McNemar’s test was applied to compare the cutoff values (10th and 90th percentiles) with the conventional cutoff values. The level of agreement between the newly defined and traditional cutoffs was determined using Cohen’s Kappa coefficient. A *p*-value of <0.05 was considered significant.

### 2.6. Ethics

This study was approved by the Research Ethical Committee of the Qassim region, Saudi Arabia (607/45/8763).

## 3. Results

**Figure 1:**
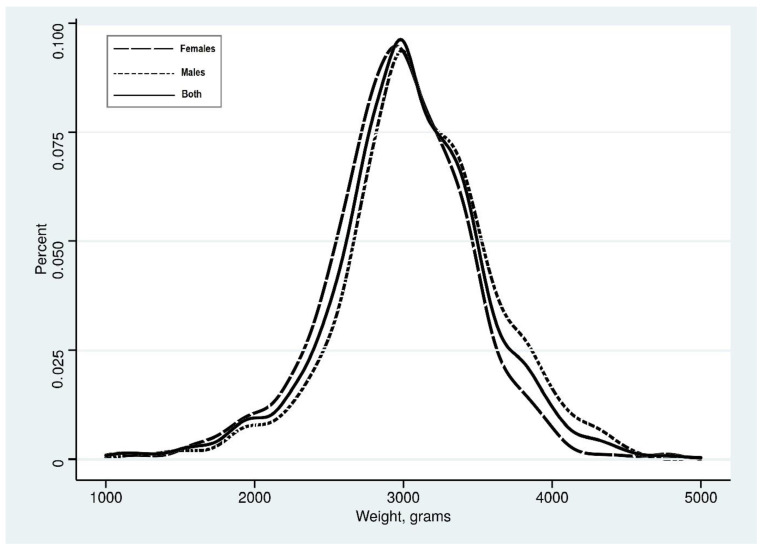
A kernel density plot of birth weight, 2022.

In this study, 1605 neonates were enrolled. The median (IQR) of the mother’s age and parity was 33 (29–37) years and 3 (2–4), respectively. Of the 1605 enrolled neonates, 828 (51.6%) were females, and 777 (48.4%) were males. The distribution of birth weight is shown in Figure 1. The median (IQR) of the birth weight was 3000 (2750–3350) g. Neonate boys’ median (IQR) birth weight was significantly higher than that of girls [3100 (2800–3400) vs. 3000 (2700–3300)] g, respectively, with *p* < 0.001.

The cutoff for LBW (the 10th percentile) in this cohort of neonates was 2450 g, while the 90th percentile defining HBW was 3650 g. Out of 1605, 157 neonates had birth weights below 2450 g (10th percentile), a prevalence of 9.8% (95.0% CI = 8.3–11.2%). The mean (95.0% CI) of the birth weight of the 157 neonates who had birth weights below 2450 g (10th percentile) was 2047 (2022–2125) g. Using the WHO (traditional) cutoff of 2500 g, 168 (10.5%) newborns had birth weights below 2500 g, and the prevalence of LBW was 10.5% (95.0% CI = 8.9–12.0%). The mean (95.0% CI) of the birth weight of the 168 neonates who had birth weights below the WHO (traditional) cutoff of 2500 g was 2098 (2048–2149) g. Thus, 11 (0.6%) newborns considered to have an LBW using the WHO cutoff (2500 g) did not have an LBW, as indicated by the 10th percentile of the current cohort. The difference between the two prevalences of LBW (using the 10th percentile and the WHO) was statistically significant (*p* < 0.001). However, as shown in Table 1, the agreement rate between the two groups was substantial (Kappa = 0.962). The cutoff for LBW (the 10th percentile) in this cohort was 2500 g for male neonates and 2400 g for females.

The cutoff to define HBW (90th percentile of birth weights) was 3650 g; 165 babies had birth weights ≥ 3650 g, a prevalence of 10.3% (95.0% CI = 8.7–11.7%). The mean (95.0% CI) of the birth weight of the 165 neonates who had birth weights ≥ 3650 g (90th percentile) was 3935 (3896–3965) g. Using a cutoff of 4000 g, 39 neonates had HBW; the prevalence of HBW was 2.4% (95.0% CI = −1.6–3.1%). The mean (95.0% CI) of the birth weight of the 39 neonates who had birth weights ≥ 4000 g was 4316 (4243–4890) g. Thus, 126 (7.8%) newborns considered to have an HBW using the cutoff (3800 g) did not have an HBW based on the 90th percentile of the current cohort. As shown in Table 1, the difference between the two prevalence rates of HBW (using the 90th percentile and the WHO) was statistically significant (*p* < 0.001). However, the agreement between the two was none or slight (kappa = 0.357). The cutoff for HBW (the 90th percentile) in this cohort of male neonates was 3800 g and 3500 g for females.

As shown in Figure 1, the range of adequate birth weight in this study population was 2450–3650 g. As shown in Table 1, the difference between the two prevalence rates of LBW (using the 10th percentile and the WHO) and the rate of agreement between male and female newborns were not significant (Kappa = 0.653).

## 4. Discussion

To our knowledge, this is the first published data estimating the cutoff points of LBW and HBW in Saudi Arabia. In this study, the 10th percentile of birth weight that defines the cutoff value of LBW was 2450 g, and the 90th percentile defining HBW was 3650 g. The current finding of the 10th percentile (2450 g) was higher than recently reported in Sudan, where the 10th percentile cutoff point was 2400 g [19]. On the other hand, the cutoff value (2450 g) of LBW in this study was lower than the previously observed values in Cameroon, where the values were 2700 and 2600 g [17,18]. The traditional birth weight cutoff of 2500 g adopted by the WHO was assumed and recommended first in Finland. Since then, it has been widely used among Western populations and the rest of the world; however, variations in birth weight continue to exist [10].

Moreover, in Mexico, it has been observed that the 10th percentile birth weight was 2530 g at the 37th week of gestational age and 2940 g at the 40th week of gestational age [23]. Likewise, higher values of 2750 g and 3000 g of the 10th percentile birth weight were reported in the United States of America [24] and Denmark, respectively [25]. The 10th percentile of birth weight appears to vary according to local sociodemographic factors and its risk profile [26]. Birth weight indicates several health parameters, maternal diseases, and perinatal parameters (e.g., maternal anemia, malaria in endemic areas, and potential risk factors for admission to the neonatal intensive unit and neonatal mortality). In sub-Saharan Africa, maternal anemia [27] and malaria [28,29], which are the leading causes of LBW, are endemic; high prevalence rates (20.0–48.0%) of anemia and malaria are reported in different countries as the leading causes of maternal morbidity.

Thus, LBW has been extensively studied in malaria and anemic endemic areas, including Africa [30]. Perhaps this explains the presence of substantial data on LBW in these countries. The other explanation for the difference in the 10th percentile of birth weight in this study and other African studies is that, in Saudi Arabia, almost all deliveries must be conducted in the hospital. In contrast, home deliveries constitute nearly half of the deliveries in Sudan [31]. This is the first study to estimate the cutoff values of LBW and HBW in a low-resource setting and the first among Sudanese newborns. Using 2500 g as the cutoff, the incidence of LBW was 10.5%. This result is lower than the findings of previous studies in different regions of Sudan [32,33,34,35]. Moreover, this incidence of LBW is also lower (13.9%) than estimates reported in Africa by the United Nations International Children’s Fund (UNICEF) and the WHO [1]. Interestingly, a high incidence (26.2%) of LBW was reported in Southeast Asia [1].

The 90th percentile birth weight that defines the cutoff value of HBW was 3650 g, and a low prevalence (2.4%) of macrosomia was detected when 4000 g was used as the cutoff for HBW. The current low prevalence (2.4%) of macrosomia was lower than the prevalence of macrosomia (19.8%) reported in a nearby area in Hail in Central Saudi Arabia using the cutoff of 4000 g [36]. A previous meta-analysis showed that the prevalence of macrosomia was highly variable in different countries, ranging from 0.5% in India to 14.9% in developing countries [37]. The current finding of the 90th percentile (3650 g) was lower than recently reported in Sudan, where the 90th percentile cutoff point was 3700 g [19]. Moreover, the reported 90th percentile of birth weight in this study was lower than the reported 90th percentile in Cameroon (3850 g) [11] and in Nigeria (3800 g) [38]. The HBW cutoff among the American population varied according to ethnicity (e.g., 4500 g was the cutoff for whites, while 4300 g was the cutoff for Blacks and Hispanics) [39]. A lower cutoff was recorded in 35,768 singleton live births in a multiethnic population in Kuwait, demonstrating that Indians-Asians had the lowest birth weights [40].

It seems that not only ethnicity but also maternal anthropometric measures (weight and height) are factors that determine birth weight [41]. Nevertheless, most studies set values for specific gestational age, aiming for high gestational age rather than HBW, regardless of gestational age [23,42,43]. It is worth noting that these different cutoff values for HBW indicate disparities in birth weight in other settings such as Cameroon (4.3−6.0%) [44,45]. Recently, when fetal growth was analyzed in 2288 pregnancies in the United States of America, using a constant coefficient, the mean and standard deviation were questioned. The calculated or customized percentiles directly via quantile regression were assumed as an alternative mathematical model for fetal growth [46].

### Limitations of This Study

Although this is the first study to address birth weight cutoff points, some limitations should be considered. First, the study only collected data representing a restricted area at Unaizah, Qassim in Saudi Arabia. Other maternity hospitals in this province might have different birth weight distributions. Therefore, it may not represent all the regions in Saudi Arabia. However, the results could provide a foundation for further research. Second, there is increased heterogeneity of ethnic backgrounds in Saudi Arabia. Conducting another study with a large sample size that analyzes birth weight in different regions of Saudi Arabia will give more precise results. We did not collect data on obstetrical outcomes, such as birth difficulties, maternal and perinatal mortality, and morbidity. Previous studies have found a correlation between birth weight and perinatal morbidity and mortality [6,11,47]. Gestational age was not considered in this study; perhaps gestational age is more important for prediction than birth weight [23]. Collecting such information for future research could explain the impact of birth weight and its cutoff on maternal and neonatal health.

## 5. Conclusions

This study identified cutoff values of 2450 g for LBW and 3650 g for HBW. Therefore, the suggested normal range for birth weight is 2450–3650 g. Children with an LBW of 2450–2500 g or an HBW of 3650–4000 g will receive unnecessary care, increasing the burden on healthcare services.

## Figures and Tables

**Table 1:** The prevalence and agreement of LBW and HBW using the 10th and 90th percentiles and the traditional methods among neonates in 2022.

Variable	Number (%)	Kappa	*p*
Prevalence of LBW using the 10th percentile (2450 g)	157 (9.8)	0. 962	<0.001
Prevalence of LBW using the WHO cutoff (2500 g)	168 (10.5)		
Prevalence of HBW using the 90th percentile (3650 g)	165 (10.3%)	0.357	<0.001
Prevalence of HBW using 4000 g as cutoff	39 (2.4)		

## Data Availability

The data presented in this study are available on request from the corresponding author. The data are not publicly available due to ethical restrictions.
